# Bilateral vocal cord paralysis after endoscopic placement of fully covered self-expandable metal stent for palliative treatment of malignant proximal esophageal obstruction: two case reports

**DOI:** 10.1186/s12876-020-01300-4

**Published:** 2020-05-14

**Authors:** Y. Chiche, G. Beltramo, T. Degand, A. Drouillard, C. Foignot, N. Baudouin, P. Bonniaud, M. Georges

**Affiliations:** 1 Department of Respiratory Diseases and Intensive Care, Reference Center for Adult Rare Pulmonary Diseases, University Hospital of Dijon – Bourgogne, Dijon, France; 2 INSERM U1231, University of Burgundy Franche-Comté, Dijon, France; 3 University of Burgundy Franche-Comté, Dijon, France; 4 Department of Hepato-Gastro-Enterology, University Hospital Dijon – Bourgogne, Dijon, France; 5 Centre des Sciences du Goût et de l’Alimentation, UMR 6265 CNRS 1234 INRA, University of Burgundy Franche-Comté, Dijon, France

**Keywords:** Vocal cord paralysis, Esophageal proximal stent, Complication

## Abstract

**Background:**

Oesophageal stents have several well-known respiratory complications, including aspiration pneumonia, fistula and airway compression. However, bilateral vocal cord paralysis has rarely been described.

**Methods:**

We describe two patients who presented with refractory dysphagia due to malignant proximal oesophageal strictures. Both received palliative treatment consisting of fully covered self-expandable metal stents that were placed across the strictures.

**Results:**

Both patients developed inspiratory stridor and acute hypoxemic respiratory failure shortly after the stent was placed. Flexible bronchoscopy revealed vocal cord paralysis in paramedian position, potentially due to extrinsic compression of the posterior branch of the recurrent laryngeal nerve following the progressive opening of the esophageal prosthesis. One patient recovered after the stent was removed.

**Conclusions:**

Bilateral vocal cord paralysis is a rare but potentially fatal complication of proximal esophagus stenting.

## Background

The respiratory complications of esophageal stents, including aspiration pneumonia, fistula and airway compression, are well described in literature [1]. However**,** bilateral vocal cord paralysis has rarely been described.

## Case presentation

### First case report

An 88-year-old patient with a history of esophageal squamous cell cancer presented with refractory dysphagia. The malignant esophageal stricture identified 18 cm from the incisors was dilated, and a fully covered Niti-S esophageal prosthesis of 80x18mm (Taewoong, Busan, South Korea) was subsequently placed across the stricture.

The day after the procedure, the patient developed inspiratory stridor and acute hypoxemic respiratory failure. X-rays of the neck showed that the stent was correctly placed. Orotracheal intubation was required on day 4. A flexible bronchoscopy showed that the vocal cords were paralyzed in paramedian position, with no evidence of locoregional edema or laryngeal injury, particularly cricoarytenoid edema or external tracheal compression. The stent was removed on day 6, and the patient was extubated on day 7. A second flexible bronchoscopy showed complete restoration of right vocal cord mobility and partial improvement on the left side. The respiratory outcome was favorable and oxygen therapy was stopped at day 10.

### Second case report

A 58-year-old patient with a history of right upper lobectomy for stage IA primary lung adenocarcinoma presented with dyspnea and aphagia. A CT scan revealed a proximal esophageal mass extending to the lower trachea with no evidence of lung cancer recurrence. A flexible bronchoscopy showed tracheal stenosis due to an apparently malignant endotracheal mass combined with external airway compression. The vocal cords were normal. A silicone tracheal prosthesis of 15 × 80 mm (Trachéobronxane, Novatech, La Ciotat, France) was placed across the stricture during rigid bronchoscopy. At the same time, gastroscopy revealed severe extrinsic esophageal compression 23 cm after the incisors, and so an esophageal prosthesis was placed (Taewoong, Busan, South Korea). Tachypnea and dyspnea appeared immediately after extubation. Flexible bronchoscopy showed that the vocal cords were in paramedian position without tracheal prosthesis migration or laryngeal edema. The patient died 6 days following the placement of the prosthesis due to acute respiratory distress.

## Discussion

**Fig. 1:**
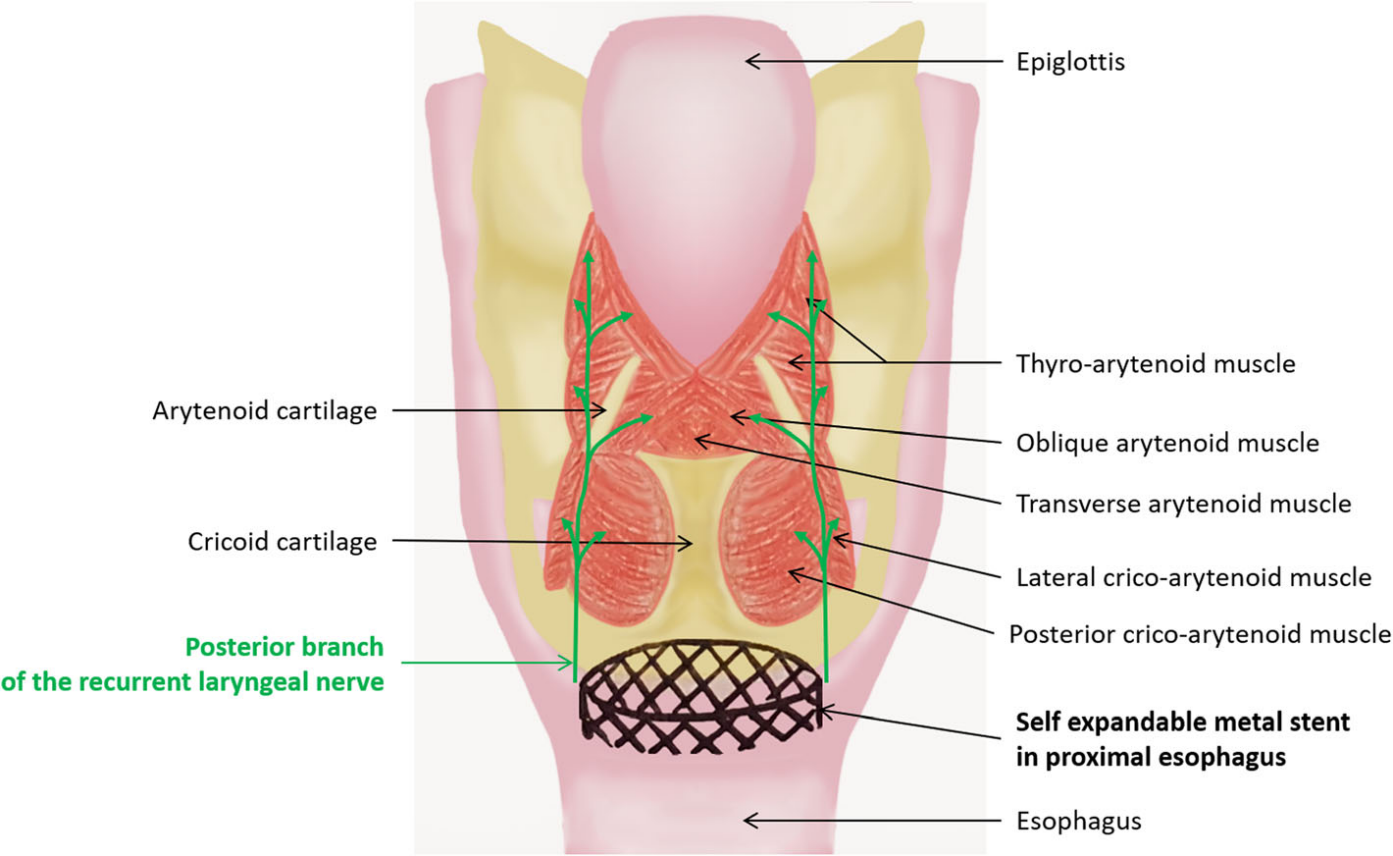
Descriptive anatomy of the larynx

The two mechanisms illustrated in Fig. 1 (adapted from Ceruse [2]) could explain the occurrence of bilateral vocal cord paralysis after the placement of proximal esophageal stents [3].

Firstly, direct compression from the esophageal prosthesis or local inflammation could induce neuropraxia of the posterior branch of the recurrent laryngeal nerve, which innervates the posterior cricoarytenoid muscles and transverse arytenoid muscles.

In addition, post-operative local inflammation may cause spasms in the interarytenoid muscles.

We presume that the progressive appearance of respiratory symptoms in our cases was due to the gradual opening of the esophageal prosthesis which increasingly compressed the posterior larynx in the days following the procedure. In previously published cases, the time to respiratory failure can vary from several hours to several days [4, 5].

Endoscopists should consider bilateral vocal cord paralysis as a rare but potentially fatal complication of proximal esophagus stenting. The diagnosis of bilateral vocal cord paralysis is a therapeutic emergency considering that patients can potentially recover once the stent is removed. Tracheostomy should also be considered as a treatment option.

## Data Availability

All data analysed during this study are available from the corresponding author on reasonable request.
